# Hospital readmissions following catheter ablation for atrial fibrillation with THERMOCOOL™ STSF/ THERMOCOOL™ ST catheter with CARTO™ 3 system versus TactiCath™ catheter with EnSite™ system

**DOI:** 10.57264/cer-2024-0075

**Published:** 2024-12-04

**Authors:** Alexandru I Costea, Rahul Khanna, Maximiliano Iglesias, Yiran Rong

**Affiliations:** 1 The Christ Hospital Cincinnati, Cincinnati, OH, USA; 2 MedTech Epidemiology & Real-World Data Science, Johnson & Johnson, New Brunswick, NJ, USA; 3 Johnson & Johnson MedTech, Franchise Health Economics & Market Access, Irvine, CA, USA

**Keywords:** arrhythmia, atrial fibrillation, CARTO 3™, catheter ablation, electroanatomical mapping, EnSite, imaging, radiofrequency catheter, TactiCath™, THERMOCOOL™ ST/STSF

## Abstract

**Aim:**

Radiofrequency (RF) catheter ablation (CA) is a mainstay treatment for atrial fibrillation (AF). RF catheters with contact force (CF) sensing technology and electroanatomical mapping systems enable real-time assessment of catheter tip-tissue interface CF, facilitating individualized and precise CA. This study examined inpatient hospital readmissions in patients with AF treated with THERMOCOOL™ ST/ THERMOCOOL™ STSF catheter with the CARTO™ 3 System versus TactiCath™ catheter with the EnSite™ System. **Materials** **&**

**methods:**

Patients undergoing CA for AF between 1 July 2019 to 30 November 2021 were identified from the Premier Healthcare Database and grouped based on use of THERMOCOOL ST/STSF or TactiCath™. Study outcomes were all-cause, cardiovascular (CV)-, and AF-related inpatient readmission at 91–365-day post-CA. Inverse probability of treatment weighting of propensity scores balanced baseline patient, comorbidity and hospital characteristics. A weighted generalized estimating equation (GEE) model examined differences in readmission outcomes.

**Results:**

A total of 15,518 patients met inclusion criteria (THERMOCOOL ST/STSF, n = 13,001; TactiCath™, n = 2517). Patient characteristics were generally well-balanced after weighting. Patients treated with THERMOCOOL ST/STSF + CARTO 3 had a 20% lower likelihood of all-cause inpatient readmissions (7.8 vs 9.3%, chi-square p = 0.041; odds ratio [OR]: 0.80, 95% confidence interval [CI]: 0.66–0.96, GEE p = 0.019) and a 21% lower likelihood of CV-related inpatient readmission (5.2 vs 6.2%, chi-square p = 0.133, OR: 0.79, 95% CI: 0.62–0.99, GEE p = 0.043) in 91–365-days post-CA versus those treated with TactiCath™ + Ensite. No significant differences were observed for AF-related readmissions.

**Conclusion:**

Patients undergoing CA for AF treated with THERMOCOOL ST/STSF + CARTO 3 had a significantly lower risk of all-cause and CV-related inpatient hospital readmission versus those treated with TactiCath™ + Ensite.

## Shareable abstract

In this study, atrial fibrillation patients who had catheter ablation procedure using the THERMOCOOL™ ST/STSF Catheter + CARTO™ 3 mapping system had significantly lower risk of readmissions versus those treated with TactiCath™ Catheter + EnSite™ system.

## Plain language summary

Hospital readmission rates after treatment for atrial fibrillation using different ablation catheters guided by electroanatomical mapping systems.

**What is this article about?:**

Atrial fibrillation is the most common cardiac rhythm disorder and is usually treated with radiofrequency ablation. The current study compared inpatient hospital readmission rates after treatment with two different catheters and electroanatomical mapping systems: THERMOCOOL™ ST/STSF Catheter using the CARTO™ 3 System (THERMOCOOL ST/STSF + CARTO 3) versus TactiCath™ Catheter using the EnSite™ System (TactiCath + Ensite).

**What were the results?:**

Patients treated with THERMOCOOL ST/STSF + CARTO 3 had a lower risk of all-cause and cardiovascular-related inpatient hospital readmissions compared with those treated with TactiCath + Ensite. There were no differences in atrial fibrillation-related inpatient readmissions between the groups.

**What do the results mean?:**

The choice of ablation catheter and electroanatomical mapping system may impact hospital readmission rates following radiofrequency catheter ablation treatment for atrial fibrillation.

## Graphical abstract

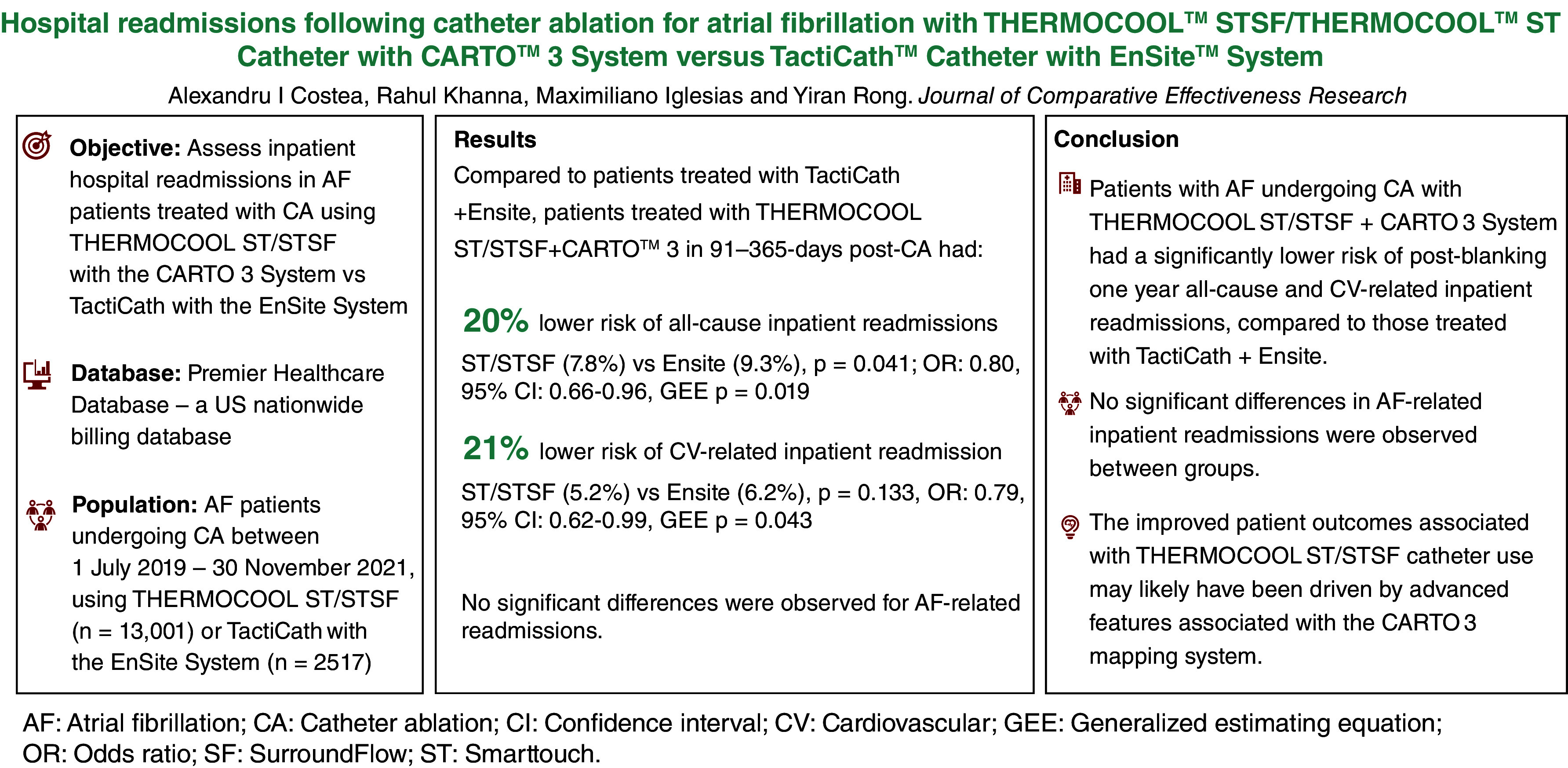

Atrial fibrillation (AF) is the most common cardiac arrhythmia in adults [1] with an estimated lifetime risk of 22–26% [2]. Over six million people in the US live with AF, and this number is projected to increase 2.5-fold by 2050. Each year, over 454,000 hospitalizations are attributed to AF [3], with numbers steadily increasing from 91.7 per 100,000 people in 1993 to 109 per 100,000 in 2014 [4]. Many of these hospitalizations are readmissions following catheter ablation (CA) treatment, with 10.5% of patients re-hospitalized within 30 days and 16.5% within 90 days [5]. Inpatient hospital costs primarily drive the financial burden of AF on the US healthcare system, which were around $26 billion in 2010 [6]. As the incidence of AF is projected to substantially rise in the coming years, there is an urgent need for cost-effective management strategies.

Catheter ablation is a cornerstone therapy for patients with AF. CA reduces AF burden, improves quality of life and reduces cardiovascular (CV) hospitalizations and death [7–10], thereby lessening healthcare utilization and associated costs among patients with AF [11]. Radiofrequency (RF) is a mainstay ablation approach that uses contact force (CF) sensing technology paired with electroanatomical mapping systems (EAMs) that guide the procedure. A meta-analysis of clinical trials and observational studies found that CF-sensing RF catheters led to a 37% reduction in AF recurrences over a 12-month period when compared with RF catheters without CF sensing technology [12]. Additionally, procedure time, fluoroscopy exposure and RF exposure are significantly reduced when using CF-sensing CA [13]. Together, RF ablation catheters with CF-sensing technology have substantially improved outcomes among patients treated for AF.

THERMOCOOL™ SMARTTOUCH (ST)/THERMOCOOL SMARTTOUCH Surroundflow (STSF) (Biosense Webster Inc., CA, USA) and TactiCath™ (Abbott, IL, USA) are two commonly used RF catheters with CF-sensing technology. THERMOCOOL™ ST/STSF uses electromagnetic location technology in a precision spring irrigated porous tip for cooling, and TactiCath uses a fiberoptic sensor tip. A single-arm trial on the safety and efficacy of VISITAG SURPOINT™-guided ablation using THERMOCOOL STSF catheter for patients with symptomatic paroxysmal AF demonstrated a low adverse event rate of 4.3% and a 92.7% freedom from AF, atrial tachycardia, or atrial flutter [14]. Similarly, the Treatment of symptomatic paroxysmal and persistent atrial fibrillation (Real-AF) study demonstrated a 2.3% of complication rate and 85.9% freedom from AF recurrence during 1-year follow-up for paroxysmal AF patients with THERMOCOOL ST or THERMOCOOL STSF [15]. Significant healthcare cost-savings have been observed in patients treated with THERMOCOOL ST/STSF. A recent retrospective cohort study found a healthcare utilization decline of 40.2% in the 12-month post CA compared with pre-CA in AF patients treated with THERMOCOOL STSF [16]. A recent single-arm trial investigating the safety and efficacy of TactiCath for patients with AF found a low adverse event rate of 4.7%, procedural success in 98% of patients and a 53% reduction in all-cause CV healthcare utilization compared with predetermined performance goals [17]. Additionally, the estimated rate of one-year free from symptomatic AF has been reported to be 82.2% and 1-year drug-free success to be 68.2% when treated with TactiCath catheter [18].

As the incidence and economic impact of AF is projected to increase in the coming years, it is important to examine patient outcomes following treatment with different RF ablation catheter devices and their associated EAM systems. However, research directly comparing these technologies is lacking. In this study, we examined all-cause, CV-related and AF-related inpatient readmissions in the post-blanking 91–365-days following CA treatment with the THERMOCOOL ST/STSF catheter versus the TactiCath catheter.

## Materials & methods

### Data source

This retrospective cohort study used hospital billing records from 1 July 2018 to 30 November 2022, obtained from the Premier Healthcare Database (PHD). The PHD includes complete clinical coding, hospital cost and patient billing data from over 1000 voluntarily participating US hospitals in its healthcare alliance. The PHD excludes federally funded hospitals (e.g., Veterans Affairs); however, the included hospitals are representative of nationwide bed sizes, geographic regions, urban and rural settings and teaching hospital status. The PHD contains a cost-accounting department log of date-stamped records of all billed items including primary and secondary diagnoses for each patients' hospitalization, medications, laboratory, diagnostic and therapeutic services.

The use of the PHD was reviewed by the New England Institutional Review Board (IRB) and determined to be exempt from broad IRB approval due to not including research on human subjects. This study adhered to the guidelines set forth by the Helsinki Declaration revised in 2013. All study reports contained aggregate data only and did not identify individual patients or physicians. Confidentiality of all patient data records was maintained at all times.

### Study sample

Adult patients who underwent an elective CA procedure with a primary diagnosis of AF (ICD-9-CM code: 427.31; ICD-10-CM codes: I48.0, I48.1, I48.2, I48.91) between 1 July 2019 and 30 November 2021 were identified. The first observed hospital admission meeting these criteria during this period was designated as the index CA. Patients were required to have undergone their index CA in a hospital that continuously provided inpatient data to the PHD during the 12-month pre-index period and post-index period. Based on the underlying catheter used during the CA procedure, patients were classified into THERMOCOOL ST/STSF or TactiCath (i.e., Tacticath, TactiCath Quartz and Tacticath SE) cohort.

Patients who had undergone a CA procedure with a primary or secondary diagnosis of AF, surgical ablation, valvular procedure, or left atrial appendage occlusion performed in the 12 months pre-index CA period were excluded. Patients were excluded if they died during the index admission and/or sex was not included in the PHD.

### Study covariates

Participant demographics assessed included age (categorized into groups: 18–49 years, 50–59 years, 60–69 years and ≥70 years), sex, race/ethnicity (white, non-white) and payer type (commercial, Medicare/Medicaid, other). Procedural characteristics assessed included the index CA year (2019, 2020, 2021) and setting (inpatient, outpatient). Participant clinical characteristics assessed included the Elixhauser Comorbidity Index based on diagnoses during the index CA (0–1, 2–3, 4+), CHA_2_DS_2_-VASc score based on diagnoses during the index CA, AF type based on the diagnosis during the index CA (Persistent AF, Paroxysmal AF), a history of cardioversion procedure or AF-related inpatient admission in the 12 months prior to the index CA and sleep apnea or obesity with a present on admission diagnosis during the index CA. Hospital characteristics assessed included the hospital size (categorized as 000–299 beds, 300–499 beds, 500+ beds), geographic location (Midwest, South, Northeast, West), hospital type (teaching, non-teaching) and ablation volume (number of admissions with ablation in the pre-index 12 months with the cut-off determined on the median value).

### Study outcomes

Study outcomes were evaluated 365 days post-index CA, following a 90-day ‘blanking period’ (days 91–365), per guidance by the Heart Rhythm Society to avoid capturing early recurrences [19]. The blanking period is defined as the first 3 months following an AF ablation as up to 50% of patients could report early recurrences of AF after catheter ablation, and these arrhythmias do not necessarily indicate therapy failure in the long-term [19]. The primary study outcome was all-cause inpatient readmissions among AF patients treated with THERMOCOOL ST/STSF + CARTO 3 versus TactiCath + EnSite™.

Secondary outcomes were post-index ablation 91–365 days CV- and AF-related inpatient readmissions among patients treated with THERMOCOOL ST/STSF + CARTO 3 versus TactiCath + EnSite.

### Statistical analysis

The inverse probability of treatment weighting (IPTW) of propensity scores approach was adopted to minimize the effect of potential confounders and balance the two cohorts on study covariates [20]. Each patient was assigned a weight by using the IPTW technique along with an estimation of the average treatment effect [20].

A logistic regression model was used to estimate the propensity score of an individual to perform the procedure using THERMOCOOL ST/STSF + CARTO 3. Then the inverse of the propensity scores were used to generate weights for each patient. Stabilized IPTW weights were calculated for each patient and applied in the analysis. The standardized mean difference (SMD) was used to determine whether the distribution of these covariates was balanced after weighting, with SMD >0.25 or <-0.25 considered an imbalanced distribution.

Descriptive statistics were reported for all study variables for both pre- and post-weighted cohort. Bivariate comparisons were conducted to examine differences in study outcomes between the two groups post-weighting, using chi-square tests. Weighted generalized estimating equation (GEE) model with logit link and binomial distribution function was used to examine differential in outcomes among study groups. GEE analyses adjusted for hospital clustering and any covariate that emerged significant (based on SMD, with values outside of the 0.25 to -0.25 range considered significant) post-weighting. For outcomes assessed in the regression analysis, the Benjamini-Hochberg (BH) correction method was used to reduce Type I errors caused by multiple comparisons [21]. *P*-values <0.05 were considered statistically significant (for BH correction, the highest *p* that was smaller than the BH critical value was identified [using false discovery rate of 0.10], with all *p*-values above the highest *p*-value then considered significant). All analyses were conducted using R software (version 4.1.2; R Foundation for Statistical Computing, Vienna, Austria).

## Results

### Patient characteristics

A total of 15,518 patients met the study criteria, with 13,001 (83.78%) treated with THERMOCOOL ST/STSF + CARTO 3 and 2517 (16.22%) treated with TactiCath + EnSite. Supplemental Figure 1 shows the study patient attrition.

Prior to IPTW, patients were balanced on sociodemographic covariates except for race and ethnicity (Table 1). The majority of patients in both groups were white (THERMOCOOL ST/STSF + CARTO 3: 84.4%; TactiCath + EnSite: 92.4%; absolute SMD [aSMD] = 0.252).

**Table 1.** Patient, procedural, clinical and hospital characteristics, pre- and post-weighting.

	Pre-weight cohort			Post-weight cohort^†^		
	THERMOCOOL™ ST/STSF (n = 13,001)	TactiCath™ (n = 2517)		THERMOCOOL™ ST/STSF	TactiCath™	
	n (%)	n (%)	aSMD	%	%	aSMD
Age (years)			0.018			0.026
18–49	898 (6.9%)	168 (6.7%)		6.9%	7.0%	
50–59	2336 (18.0%)	440 (17.4%)		18.1%	18.7%	
60–69	4706 (36.2%)	928 (36.9%)		36.4%	36.9%	
≥70 years	5061 (38.9%)	981 (39.0%)		38.6%	37.4%	
Sex			0.058			0.050
Male	8294 (63.8%)	1675 (66.5%)		64.3%	66.6%	
Female	4707 (36.2%)	842 (33.5%)		35.7%	33.4%	
Race			**0.252**			0.154
White	10,969 (84.4%)	2325 (92.4%)		85.4%	90.4%	
Non-White	2032 (15.6%)	192 (7.6%)		14.6%	9.6%	
Payer type			0.045			0.069
Medicare or Medicaid	7849 (60.4%)	1546 (61.4%)		60.0%	58.0%	
Commercial	4673 (35.9%)	898 (35.7%)		36.4%	39.2%	
Other	479 (3.7%)	73 (2.9%)		3.6%	2.8%	
Elixhauser comorbidity index			0.181			0.027
0–1	1909 (14.7%)	528 (21.0%)		15.2%	15.4%	
2–3	6213 (47.8%)	1194 (47.4%)		47.8%	48.9%	
≥4	4879 (37.5%)	795 (31.6%)		37.0%	35.7%	
CHA_2_DS_2_-VASc			0.062			0.035
0–1	3580 (27.5%)	753 (29.9%)		28.0%	29.2%	
2–3	6357 (48.9%)	1222 (48.6%)		48.8%	48.9%	
≥4	3064 (23.6%)	542 (21.5%)		23.2%	21.9%	
Index year			0.046			0.029
2019	2680 (20.6%)	475 (18.9%)		20.7%	21.7%	
2020	4831 (37.2%)	971 (38.6%)		37.4%	37.7%	
2021	5490 (42.2%)	1071 (42.5%)		41.9%	40.6%	
Setting			0.131			0.012
Inpatient	1171 (9.0%)	141 (5.6%)		8.8%	8.5%	
Outpatient	11,830 (91.0%)	2376 (94.4%)		91.2%	91.5%	
Sleep apnea	3562 (27.4%)	721 (28.6%)	0.028	27.9%	31.4%	0.076
Obesity	3163 (24.3%)	529 (21.0%)	0.079	24.4%	25.7%	0.031
Cardioversion	2300 (17.7%)	518 (20.6%)	0.073	18.1%	20.7%	0.064
AF-related IP admission	1238 (9.5%)	180 (7.2%)	0.086	9.4%	9.1%	0.009
AF type			0.022			0.035
Persistent AF	6545 (50.3%)	1295 (51.5%)		50.3%	48.5%	
Paroxysmal AF	6456 (49.7%)	1222 (48.5%)		49.7%	51.5%	
Hospital bed size			**1.155**			**0.306**
<300	491 (3.8%)	928 (36.9%)		6.2%	10.6%	
300–499	2776 (21.3%)	857 (34.0%)		24.4%	34.5%	
≥500	9734 (74.9%)	732 (29.1%)		69.4%	54.9%	
Hospital region			**1.152**			**0.381**
Midwest	2287 (17.6%)	602 (23.9%)		19.4%	28.2%	
Northeast	2288 (17.6%)	72 (2.9%)		15.6%	6.1%	
South	7714 (59.3%)	765 (30.4%)		56.5%	52.5%	
West	712 (5.5%)	1078 (42.8%)		8.5%	13.2%	
Hospital type			**0.574**			0.104
Teaching	9343 (71.9%)	1124 (44.7%)		68.6%	63.7%	
Non-teaching	3658 (28.1%)	1393 (55.3%)		31.4%	36.3%	
Ablation volume			**0.304**			**0.457**
**≥**330	6839 (52.6%)	948 (37.7%)		48.2%	26.6%	
<330	6162 (47.4%)	1569 (62.3%)		51.8%	73.4%	

† 95% percentile truncate method was applied. Average treatment effect estimate was conducted.

Bold aSMD values represent significant difference, as they are outside the 0.25 to -0.25 range.

THERMOCOOL™ ST/STSF = THERMOCOOL SMARTTOUCH™/THERMOCOOL SMARTTOUCH™ Surroundflow.

AF: Atrial fibrillation; aSMD: Absolute value for standardized mean difference.

The majority of patients in the THERMOCOOL ST/STSF + CARTO 3 group were treated in hospitals with ≥500 beds (74.9%, [9734/13,001]), whereas most patients in the TactiCath + EnSite group were treated in hospitals with <300 beds (36.9%, [928/2517]; aSMD = 1.155). The majority of patients in the THERMOCOOL ST/STSF + CARTO 3 group were treated in hospitals located in the South (59.3%, [7714/13,001]), whereas most patients in the TactiCath + EnSite group were treated in hospitals located in the West (42.8%, [1078/2517]; aSMD = 1.152). The majority of patients in the THERMOCOOL ST/STSF + CARTO 3 group were treated in teaching hospitals (71.9%, [9343/13,001]), whereas most patients in the TactiCath + EnSite group were treated in non-teaching hospitals (55.3%, [1393/2517]; aSMD = 0.574). The majority of patients in the THERMOCOOL ST/STSF + CARTO 3 group were treated in a hospital with an ablation volume ≥330 (52.6%, [6839/13,001]), whereas most patients in the TactiCath + EnSite group had their procedure in a hospital with an ablation volume <330 (62.3%, [1569/2517]; aSMD = 0.304). After weighting, the study cohorts were well balanced on all covariates except hospital bed size, hospital region and ablation volume. To alleviate any potential bias that may could be introduced due to significant differences in these three covariates (in the post-weighted sample), these three covariates were then adjusted for in the GEE model to control for any influence of these covariates on study outcomes. Table 1 lists the patient demographic, procedural, clinical and hospital characteristics pre-and post-weighting.

### Outcomes

All-cause inpatient readmissions 91–365-day post-index CA were significantly lower for patients treated with THERMOCOOL ST/STSF + CARTO 3 compared with TactiCath + EnSite in the bivariate analysis (7.8% vs 9.3%, p = 0.041) for the weighted cohort. There were no significant differences in bivariate analysis for 91–365 days CV-related (5.2% vs 6.2%, p = 0.133) and AF-related (3.2% vs 3.7%, p = 0.358) inpatient readmissions among THERMOCOOL ST/STSF + CARTO 3 versus TactiCath + EnSite cohort. Results from GEE analyses revealed a 20% lower risk of 91–365 days post-CA all-cause inpatient readmissions among AF patients treated with THERMOCOOL ST/STSF + CARTO 3 compared with TactiCath + EnSite (odds ratio [OR]: 0.80, 95% confidence interval [CI]: 0.66–0.96, p = 0.019). Patients treated with THERMOCOOL ST/STSF + CARTO 3 also had 21% lower risk of CV-related inpatient readmissions in the 91–365 days post-CA period as compared with those treated with TactiCath + EnSite (OR: 0.79, 95% CI: 0.62–0.99, p = 0.043). GEE analyses did not reveal any significant differences in AF-related inpatient readmissions among patients treated with THERMOCOOL ST/STSF + CARTO 3 versus TactiCath + EnSite (Table 2).

**Table 2.** Hospital readmission outcomes in the 91–365 day post-index period.

	THERMOCOOL™ ST/STSF^#^	TactiCath™^#^	Chi-square test, p-value	GEE regression model^§^, OR	GEE regression model, 95% CI	GEE regression model, p-value^¶^
All-cause IP readmission	7.8%	9.3%	**0.041**	0.80	0.66–0.96	**0.019**
CV-related IP readmission^†^	5.2%	6.2%	0.133	0.79	0.62–0.99	**0.043**
AF-related IP readmission^‡^	3.2%	3.7%	0.358	0.80	0.59–1.07	0.134

† Cardiovascular (CV)-related inpatient readmission was defined as inpatient admission with CV conditions as the primary diagnosis in the 91–365 days post-index CA period.

‡ AF-related inpatient readmission was defined as inpatient admission with AF as the primary diagnosis in the 91–365 days post-index CA period.

§ Hospital bed size, hospital region and ablation volume in 12-months prior to index for the index CA hospital were controlled in the GEE regression models.

¶ Benjamini Hochberg (BH) critical values were: 0.033 for all-cause readmissions; 0.067 for CV-related readmissions; and 0.100 for AF-related readmissions. As such, the results after BH correction remained consistent and indicated significant differences for all-cause and CV-related inpatient readmissions.

# Rates for the weighted cohorts.

Bold p-values represent significant findings.

AF: Atrial fibrillation; CI: Confidence interval; CV: Cardiovascular; IP: Inpatient; OR: Odds ratio; THERMOCOOL™ ST/STSF: THERMOCOOL SMARTTOUCH™/THERMOCOOL SMARTTOUCH™ Surroundflow.

## Discussion

In this retrospective cohort study, AF patients undergoing CA using THERMOCOOL ST/STSF + CARTO 3 had significantly lower risk of all-cause and CV-related inpatient readmissions compared with patients treated with TactiCath + EnSite. Specifically, patients were 20% less likely to have all-cause inpatient readmissions and 21% less likely to have a CV-related inpatient readmissions when treated with THERMOCOOL ST/STSF + CARTO 3. No significant differences in AF-specific inpatient readmissions were observed between groups.

Patients in the current study were grouped based on the type of catheter used during CA. Both catheters use CF-sensing technology but with different mechanisms. THERMOCOOL ST/STSF has an irrigated porous tip with an embedded precision spring to measure CF, while TactiCath catheters have a fiberoptic CF sensor embedded in an irrigated tip. The THERMOCOOL STSF has established features including dual compression coil design for enhanced deflection and torque thereby leading to improved catheter handling. Further, the presence of CF vector and deflection indicators on the catheter tip allows for enhanced assessment of directionality of CF in the THERMOCOOL STSF device. The use of THERMOCOOL ST/STSF has been shown to be associated with significant cost savings [16,22,23], and good short- and long-term safety and clinical success rates [14,15,18,24–28]. In addition to different catheter features, advanced EAMs such as CARTO 3 and EnSite allow for individualized diagnostic and mapping by tracking intracardiac electrodes in three-dimensional maps of the heart to locate the origin of the arrhythmia [29]. By recording electrical information with mapping catheters, EAMs produce a non-fluoroscopic three-dimensional geometrical reconstruction with a color-coded display of the electrical activation sequence, called ‘activation mapping’, providing a highly targeted treatment region. The integration of advanced EAMs in CA procedures have improved patient outcomes by reducing procedural time, radiation exposure [30,31] and improved CA success [32], making them a mainstay in RF treatment for AF. To visualize the RF catheter tip, CARTO 3 uses current- and magnetic-based technology for catheter visualization and location accuracy, while EnSite uses impedance measurement technology to visualize the catheters and this impedance data cannot simultaneously incorporate with the magnetic-based data that TactiCath catheters generate for visualization [29]. While both EAMs allow for high-density, real-time assessment of wall contact, catheter stability, anatomical position and relationship between the ablation catheter and mapping catheter [33], differences in software capabilities between the two systems may ultimately impact patient outcomes.

The ability to maintain stable CF during CA directly impacts the success of the procedure. Both systems, CARTO 3 and EnSite have software modules to provide feedback on the lesions being performed while ablating. The Lesion Size Index (LSI) implementation in the EnSite system is a proprietary index incorporating time, power, CF and impedance. In 2018, the VISITAG SURPOINT Module was integrated into CARTO 3 (Biosense Webster, Inc., CA, USA) to optimize ablation phase by eliminating variability and improving accuracy. This software provides a visual real-time indication of the progress of the customized ablation strategy through an objective index tagging. The index is a result of a weighted exponential formulas that takes in account power, CF and RF application time. Several studies have demonstrated improvements in procedures and patient outcomes using this software. A meta-analysis of data from 11 studies demonstrated that RF ablation using VISITAG SURPOINT had a significantly higher first-pass isolation rate, lower acute pulmonary vein reconnection rate and fewer arrhythmia relapses [34]. Two large prospective studies with >600 patients at 49 different hospitals treated with this software demonstrated improvement in patient outcomes including a 90% freedom from tachyarrhythmia recurrence in 12 months, ≥90% freedom from repeat ablation rate, and a low primary adverse event rate of ∼4% while having a clinically meaningful improvement in quality of life [35–38]. When comparing use of this software to non-use, a 50% decrease in acute reconnections and 22% increase in 12-month freedom from AF recurrence without anti-arrhythmic drug use was reported [39]. Though difficult to confirm, the VISITAG SURPOINT Module software, unique to CARTO 3, may have contributed to better patient outcomes in this study following treatment with THERMOCOOL ST/STSF + CARTO 3.

As such, the reduced risk of hospital readmissions observed in patients treated with THERMOCOOL ST/STSF + CARTO 3 in our study could potentially be attributed to the uniqueness of features for CARTO 3 mapping system and THERMOCOOL ST/STSF catheters. As EAM software continues to evolve, future studies should directly compare the impact of different systems on patient outcomes and evaluate cost-effective and safe management strategies for AF. Our findings may help guide clinical decision-making that can reduce healthcare utilization and costs for patients managing AF.

### Limitations

This study has a few limitations. In PHD, readmissions can only be assessed if the patient were to visit the same hospital (where the index procedure was performed). It is likely that the rate of readmission reported in our study is an undercount of the true rate. However, it should be noted that this limitation is likely to cause non-differential bias, thereby not influencing directionality of results observed. In addition, the device search strategy was based on combination of device name and catalog identifiers, which may have led to misidentification. Until unique device identifiers (UDIs) are universally adopted and listed, our search strategy offers a reasonable approach for device identification. Further, it should be noted that our device search was based on the underlying catheter (THERMOCOOL ST/STSF and TactiCath [including Tacticath, TactiCathQuartz, and Tacticath SE]) and not on the software system. Moreover, information on important ablation parameters such as ablation technique, procedure time and AF severity were not listed in the database, and could not be controlled for in our analyses. Further, we could not control for differential in physician experience; however, to potentially alleviate any such impact, we controlled for hospital ablation volume in our study. Though PHD includes one in five inpatient admissions, the device information is not available for every CA procedure in PHD database. Therefore, our results may not be fully generalizable to all AF patients undergoing ablation procedure using the device under investigation.

## Conclusion

Patients with AF undergoing CA with THERMOCOOL ST/STSF catheter had a significantly lower risk of post-blanking one year all-cause and CV-related inpatient readmissions as compared with those treated with TactiCath catheter (i.e., Tacticath, TactiCath Quartz and Tacticath SE). No statistically significant differences in AF-related inpatient readmissions were observed between groups. The improved patient outcomes associated with THERMOCOOL ST/STSF use may likely have been driven by advanced features associated with the CARTO 3 mapping system.

## Summary points

- We conducted a retrospective cohort study investigating 91–365-day post-ablation inpatient hospital readmissions following use of THERMOCOOL™ ST/STSF + CARTO™ 3 versus TactiCath™ + EnSite™ for the treatment of atrial fibrillation.
- We analyzed data from 15,518 patients using the PHD, a database containing complete clinical coding, hospital cost and patient billing data from over 1000 US hospitals representative of nationwide bed sizes, geographic regions, locations and teaching hospital status.
- Patients were well-balanced on baseline demographic, procedural and clinical characteristics after inverse probability of treatment weighting of propensity scores.
- Regression results showed a 20% lower risk of 91–365-day all-cause inpatient readmissions and 21% lower risk of cardiovascular-related inpatient admissions for the THERMOCOOL ST/STSF + CARTO 3 group versus the TactiCath + EnSite group. No statistically significant differences in AF-related inpatient readmissions were observed between the two groups.
- Study results highlight the significant improvements in patient outcomes associated with the use of THERMOCOOL ST/STSF catheter. Besides catheter differences, these improvements are likely accrued due to advanced features of CARTO 3 mapping system as compared with the EnSite system.

## Supplementary Material


