# Supplementary material for: Hospital readmissions following catheter ablation for atrial fibrillation with THERMOCOOL™ STSF/ THERMOCOOL™ ST catheter with CARTO™ 3 system versus TactiCath™ catheter with EnSite™ system
Source: J Comp Eff Res. 2024 Dec 4;14(1):e240075. doi: 10.57264/cer-2024-0075 (PMC11656344; doi:10.57264/cer-2024-0075)
Supplement: Supplementary file 1 [file cer-14-240075-s1.docx]

**Supplemental Material**

**Supplemental Figure 1. Patient attrition.**

Adult patients who underwent CA procedure with a primary diagnosis of AF in any setting between Jul 2019 – Nov 2021 (with first CA procedure ***as index***)

N = 53,287

Exclude patients who had unknown sex information

N = 15,518

Exclude patients who died during the index admission for CA

N = 15,520

Exclude patients who had undergone CA, surgical ablation, valvular procedure or left atrial appendage occlusion during the 12-month pre-index period

N = 15,528

Patients who had index hospitalization at hospitals that contributed inpatient data to PHD database during the 12-month pre- and post-index period (to ensure a relatively complete patients’ data in the study period)

N = 16,130

Patients with index admission criteria as ‘elective’ (to exclude the patients who underwent index CA in an urgent or emergency setting)

N = 19,441

Patients who had a clear catheter information during index CA and using either THERMOCOOL SMARTTOUCH™ (ST), THERMOCOOL™ STSF, or TactiCath™ catheter

N = 22,325

THERMOCOOL™ ST/STSF+CARTO™ 3cohort

N = 13,001

TactiCath™+Ensite™ cohort

N = 2,517
